# Conventional and complementary health care use and out-of-pocket expenses among Australians with a self-reported mental health diagnosis: a cross-sectional survey

**DOI:** 10.1186/s12913-021-07162-0

**Published:** 2021-11-23

**Authors:** Erica McIntyre, Tracey Oorschot, Amie Steel, Matthew J Leach, Jon Adams, Joanna Harnett

**Affiliations:** 1 Australian Research Centre in Complementary and Integrative Medicine, School of Public Health, Faculty of Health, University of Technology Sydney, 2007 Ultimo, NSW Australia; 2 Institute for Sustainable Futures, University of Technology Sydney, PO Box 123, 2007 Ultimo, NSW Australia; 3 National Centre for Naturopathic Medicine, Southern Cross University, NSW 2480 Lismore, Australia; 4 Faculty of Medicine and Health, The University of Sydney School of Pharmacy, 2006 Sydney, NSW Australia

**Keywords:** Mental Health, Health Services, Health Care expenses, Complementary medicine, Survey

## Abstract

**Background:**

Mental health disorders are a global health concern. In Australia, numerous national reports have found that the current mental healthcare system does not adequately meet the needs of Australians with mental illness. Consequently, a greater understanding of how people with a mental health disorder are using the broader healthcare system is needed. The aim of this paper is to explore conventional and complementary health care use and expenditure among Australian adults reporting a mental health disorder diagnosis.

**Methods:**

A cross-sectional online survey of 2,019 Australian adults examined socio-demographic characteristics, complementary and conventional health care use and the health status of participants.

**Results:**

32 % (*n* = 641) of the total sample (*N* = 2019) reported a mental health disorder in the previous 3 years. Of these, 96 % reported consulting a general practitioner, 90.6 % reported using prescription medicines, 42.4 % consulted a complementary medicine practitioner, 56.9 % used a complementary medicine product and 23 % used a complementary medicine practice. The estimated 12-month out-of-pocket health care expenditure among Australians with a mental health disorder was AUD$ 4,568,267,421 (US$ 3,398,293,672) for conventional health care practitioners and medicines, and AUD$ 1,183,752,486 (US$ 880,729,891) for complementary medicine practitioners, products and practices. Older people (50–59 and 60 and over) were less likely to consult a CM practitioner (*OR* = 0.538, 95% CI [0.373, 0.775]; *OR* = 0.398, 95% CI [0.273, 0.581] respectively) or a psychologist/counsellor (*OR* = 0.394, 95% CI [0.243, 0.639]; *OR* = 0.267, 95% CI [0.160, 0.447] respectively). People either looking for work or not in the workforce were less likely to visit a CM practitioner (*OR* = 0.298, 95% CI [0.194, 0.458]; *OR* = 0.476, 95% CI [0.353, 0.642], respectively).

**Conclusions:**

A substantial proportion of Australian adults living with a mental health disorder pay for both complementary and conventional health care directly out-of-pocket. This finding suggests improved coordination of healthcare services is needed for individuals living with a mental health disorder. Research examining the redesign of primary health care provision should also consider whether complementary medicine practitioners and/or integrative health care service delivery models could play a role in addressing risks associated with complementary medicine use and the unmet needs of people living with a mental health disorder.

## Background

Mental health disorders (MHD) are a global health concern, with as many as one in three people worldwide, and one in five Australians, reporting a common MHD (depression, anxiety or substance use disorder) in the past 12 months [1, 2]. These disorders are one of the largest contributors of global disease burden, representing 32 % of years lived with disability from all causes [3]. In addition, mental health disorders are associated with lower social and economic participation, diminished physical health and subsequent chronic illness, lower standards of living and premature death [4]. Economic costs, particularly out-of-pocket (OOP) costs, further contribute to the burden of mental illness by increasing financial pressures, which in turn, contribute to health inequities [5, 6].

Despite the considerable burden of MHD, there has been little evidence of improvement in mental health outcomes in many countries, including Australia [4]. The Australian Productivity Commission’s (APC) Inquiry Report [4] recognises that the current mental health care system does not adequately meet the needs of Australians with mental illness and has called for substantial reforms to drive significant change in how mental ill-health is understood and treated. These unmet health needs may, in part, drive many people with mental health problems to use a variety of health care services and interventions [4, 7], including complementary medicine (CM) services, products and practices that are not considered part of mainstream health care [7–9]. Previous research has revealed that almost 75 % of Australian adults experiencing anxiety report using herbal medicines (a type of CM product) in their lifetime, with relatively fewer (55 %) using prescribed medications [10]. In people with depressive disorders, it is estimated that up to 30 % of adults use CM [9].

Given the high rates of CM use among people living with mental illness, it is not surprising that OOP costs related to CM use have been shown to be higher than that reported for conventional pharmaceutical use [6]. A recent whitepaper by Zurich Australia [11] estimated individual OOP costs specific to mental health care to be close to AUD$ 1,350 (US$ 1,004) per annum. Consistent with mental health care expenditure in general, OOP costs are continuing to rise, which may result in sub-standard care or avoidance of care—both contributing to poor mental health outcomes [5, 11]. As these estimates are limited to conventional health care use, it is likely that OOP costs will be substantially greater when CM use is taken into account.

The multiple factors influencing mental health outcomes, discussed above, have important policy implications. In fact, Spinks and Hollingsworth [6] have called for government action to integrate CM-related issues into policy, not dissimilar to policies concerning conventional health care use. The reasons for this call for action are clear. CM users with mental illness are more inclined to self-treat and less likely to seek professional treatment [6]. In addition, concurrent CM and conventional medicine use [6, 7], combined with non-disclosure and poor health literacy, creates myriad health risks (e.g., delayed, inappropriate or harmful treatment) that are yet to be meaningfully addressed at the policy level [6, 7, 12].

To address policy gaps pertinent to health care use and their impacts, it is imperative to understand how people with a MHD use health services, products and practices. Alarmingly, there has not been an investigation of conventional and CM health care use in adults with a MHD in Australia in over a decade. This study addresses this knowledge gap by examining conventional and CM health care use, including OOP costs, among Australian adults reporting a MHD as well as exploring the sociodemographic and health-related characteristics of health service users, and the prevalence and predictors of health care use in this population.

## Methods

### Study design

National cross-sectional survey.

### Setting and participants

This study used a sample (*N* = 2,025) principally representative of the Australian population with regards to age, gender and state/territory of residence as indicated by Australian Bureau[13] of Statistics data [14]. For a comparison between this sample and the national population data see Steel et al. [13]. Employing a cross-sectional design, the reported results drew from a sub-study analysis of a wider project. Data collection and recruitment was conducted between 26 July and 28 August 2017, by contacting registered participants of Qualtrics (an online platform used for research participant recruitment).

Adopting a purposive convenience sampling method, eligible Australian participants aged 18 years and over, were contacted by email (*N* = 41,000) and instructed to use the weblink provided to obtain further information about the study. The response rate was 5 %. Respondents indicating an interest to participate were asked to provide consent prior to commencing the online survey. All respondents who completed the survey confirmed their informed consent. The completion time of the survey was approximately 15 min. Reflecting the financial incentive scheme of registered Qualtrics members, a small financial reward was offered to participants who completed the survey.

### Measures

The 50-item survey included questions concerning socio-demographic characteristics, health care use and health status. Using a convenience sample of seven Australian adults, the survey items were tested with respect to response option suitability, question construction and ease of use. Respondent feedback resulted in minor changes to the questionnaire.

### Health status

Participants were asked if they had been diagnosed with any health conditions within the last 3 years (yes/no) from a list of chronic health conditions, including the following mental health diagnoses: mood disorder (e.g., depression), anxiety disorder, sleep disorder, substance use disorder, schizophrenia or other psychotic disorder, or other mental health disorder.

### Health care use

Participants were asked about their conventional health care use within the last 12 months including consultation with a primary care physician, allied health worker (e.g., counsellor), medical specialist, or hospital-based health care professional (e.g., hospital doctor). The 20-item Short Form Health Survey (SF-20) was used to assess health related quality of life across six dimensions of health: physical functioning, role functioning, social functioning, mental health (MHI-5), current health perceptions, and pain [15]. The five items comprising the MHI-5 measure the frequency of general mental health symptoms in the previous month on a 6-point scale (1 = All of the time to 6 = None of the time), with higher summed scores indicating better mental health. Participants were also asked to report consultation frequency, reason for consultation and estimated OOP costs.

CM use was measured using items from the validated International Complementary and Alternative Medicine Questionnaire (I-CAM-Q) [16, 17]. As the I-CAM-Q requires country specific adaptation [18], some changes were made to reflect Australian cultural contexts. Participants were asked about their CM usage within the last 12 months with respect to CM products, mind-body practices and consultations with CM practitioners. Reasons for these consultations and estimated OOP costs were also solicited.

### Data analysis

IBM SPSS Statistics Premium Edition Version 25 (Armonk, New York, IBM Corp) was used to analyse the data. Prior to conducting analyses, raw data were screened for any missing or incomplete responses. During this process, six cases were removed as the data were deemed unreliable; this included text responses that were incongruous with the corresponding question, repeated patterns throughout the data, and inconsistency in responses. After removal of the six cases, 2,019 cases were included in the final data set. Binary variables were created from categorical variables that related to CM use, as well as diagnosed illnesses. Mental health diagnoses were categorised into four groups for analysis: any MHD, anxiety disorder, mood disorder, and other MHD (all excluding anxiety and mood). The total summed score of the MHI-5 items was transformed a standard score from to 0 to 100. Descriptive statistics were used to determine the percentages and frequencies. Chi-square analysis was used to assess associations between categorical sociodemographic and health-related variables. Cramer’s V was used to measure the magnitude of these associations. Independent t-tests were used to determine differences between groups for continuous variables; the Welch t-test was used if the homogeneity of variance assumption was violated according to Levene’s test (*p* > .05). Covariates were included in regression analyses if they were theoretically relevant or had an association *p *< .25 [19]. Logistic regression was used to identify predictors of health care use. Hierarchical multiple regression was used to predict total health care expenditure from MHD. Statistical significance was set to *p* < .05.

Participants’ mean OOP expenditure for health services and medicines—inclusive of CM and conventional health—was used for the economic analysis. As the economic data were not normally distributed, Mann-Whitney U tests were used to determine the median differences in OOP expenses (continuous variable) between those with and without a MHD (categorical variable). Population level OOP expenditure was calculated for the estimated number of the Australian population with a MHD—determined based on the prevalence of MHD reported in this study multiplied by the number of Australian adults aged 20 years or above (*n* = 17, 615, 676) reported in Australian census figures for 2016 [20]. Data for the number of Australian adults 18 to 19 years was not available. The estimated total expenditure by Australian population with a MHD was then extrapolated by multiplying the reported mean expenditure by MHD participants in this study by the estimated number of Australians with a MHD. Expenses are reported in Australian dollars.

### Ethics

 The Human Research Ethics Committee at Endeavour College of Natural Health provided ethics approval (20,170,242) in accordance with the National Health and Medical Research Council Statement on Ethical Conduct in Human Research. Charles Sturt University (H17048), The University of Sydney (2017/140) and The University of Technology Sydney (ETH17-1564) Human Research Ethics Committees also granted ethics approval.

## Results

32 % (*n* = 641) of the total sample (*N* = 2019) reported a MHD in the previous 3 years. Of these, 20 % (*n* = 402) reported an anxiety disorder, 19 % (*n* = 387) a mood disorder and 18 % (*n* = 372) at least one other MHD. The general mental health status for the total sample (*M* = 63.63, *SD* = 22.98) was slightly lower than previously reported general population means [21]. The mental health status of those reporting a mood disorder (*M* = 42.94, *SD* = 20.54), anxiety disorder (*M* = 42.97, *SD* = 20.82) or other MHD (*M* = 46.04, *SD* = 22.47) was consistent with clinical population norms [22].

### Sociodemographic characteristics

Table 1 presents the descriptive statistics for the participants sociodemographic characteristics and their association with each category of MHD (i.e., any MHD, anxiety disorder, mood disorder, and other MHD). As detailed in Table 1, there were statistically significant associations (*p* < .05) between gender, employment status, marital status, educational qualification, financial manageability, health care card or private health insurance and all MHD categories. The magnitude of these associations were mostly small, with Cramer’s V values ranging between 0.06 and 0.25. Those who were female (compared to males), not in the paid workforce (compared to those in the workforce), never married (compared to those who are or have been married), or had a vocational qualification (compared to those with an education level at or below year 12) more frequently reported all MHD categories. Participants with a health care card, compared to those without, reported all categories of MHD less frequently. In contrast, those who found financial management difficult some or all of the time (compared to those finding it not too bad or easy) and those with private health insurance (compared to those without) more frequently reported all categories of MHD.

**Table 1:** Sociodemographic characteristics of participants with and without a mental health disorder diagnosis

Sociodemographic show characteristic	Any mental health disorder n (%)			Anxiety disorder n (%)			Mood disorder n (%)			Other mental health disorder n (%)		
	No (*n* = 1378)	Yes (*n* = 641)	*P*-value (Cramer’s V)	No (*n* = 1617)	Yes (*n* = 402)	*P*-value (Cramer’s V)	No (*n* = 1632)	Yes (*n* = 387)	*P*-value (Cramer’s V)	No (*n* = 1647)	Yes (*n* = 372)	*P*-value (Cramer’s V)
*Gender*			< 0.001* (0.14)			< 0.001* (0.18)			< 0.001* (0.15)			0.042* (0.05)
Female	639 (46.4)	395 (61.7)		758 (46.9)	276 (68.8)		778 (47.7)	256 (66.3)		826 (50.2)	208 (56.1)	
Male	737 (53.6)	245 (38.3)		857 (53.1)	125 (31.2)		852 (52.3)	130 (33.7)		819 (49.8)	163 (43.9)	
*Age (years)*			0.026* (0.07)			< 0.001* (0.15)			0.005* (0.09)			0.210
18–29	348 (25.3)	164 (25.6)		388 (24)	124 (30.8)		414 (25.4)	98 (25.3)		419 (25.4)	93 (25)	
30–39	205 (14.9)	108 (16.8)		242 (15)	71 (17.7)		246 (15.1)	67 (17.3)		246 (14.9)	67 (18)	
40–49	235 (17.1)	127 (19.8)		268 (16.6)	94 (23.4)		287 (17.6)	75 (19.4)		289 (17.5)	73 (19.6)	
50–59	202 (14.7)	104 (16.2)		251 (15.5)	55 (13.7)		233 (14.3)	73 (18.9)		248 (15.1)	58 (15.6)	
60 and over	388 (28.2)	138 (21.5)		468 (28.9)	58 (14.4)		452 (27.7)	74 (19.1)		445 (27)	81 (21.8)	
*ARIA+*			0.007* (0.07)			0.020* (0.06)			0.018* (0.06)			0.101
Major cities	1027 (74.5)	445 (69.4)		1197 (74.0)	275 (68.4)		1211 (74.2)	261 (67.4)		1217 (73.9)	255 (68.5)	
Inner regional	227 (16.5)	143 (22.3)		277 (17.1)	93 (23.1)		281 (17.2)	89 (23)		289 (17.5)	81 (21.8)	
Outer regional/remote	124 (9)	53 (8.3)		143 (8.8)	34 (8.5)		140 (8.6)	37 (9.6)		141 (8.6)	36 (1.8)	
*State/Territory*			0.613			0.344			0.924			0.427
ACT	18 (1.3)	11 (1.7)		19 (1.2)	10 (2.5)		22 (1.3)	7 (1.8)		24 (1.5)	5 (1.3)	
New South Wales	416 (30.2)	181 (28.2)		485 (30)	112 (27.9)		483 (29.6)	114 (29.5)		494 (30)	103 (27.7)	
Northern Territory	3 (0.2)	2 (0.3)		3 (0.2)	2 (0.5)		3 (0.2)	2 (0.5)		3 (0.2)	2 (0.5)	
Queensland	313 (22.7)	151 (23.6)		366 (22.6)	98 (24.4)		373 (22.9)	91 (23.5)		380 (23.1)	84 (22.6)	
South Australia	131 (9.5)	57 (8.9)		146 (9)	42 (10.4)		151 (9.3)	37 (9.6)		147 (8.9)	41 (11)	
Tasmania	33 (2.4)	16 (2.5)		40 (2.5)	9 (2.2)		40 (2.5)	9 (2.3)		42 (2.6)	7 (1.9)	
Victoria	319 (23.1)	169 (26.4)		393 (24.3)	95 (23.6)		395 (24.2)	93 (24)		388 (23.6)	100 (26.9)	
Western Australia	145 (10.5)	54 (8.4)		165 (10.2)	34 (8.5)		165 (10.1)	34 (8.8)		169 (10.3)	30 (8.1)	
*Employment status*			< 0.001* (0.18)			< 0.001* (0.15)			< 0.001* (0.16)			< 0.001* (0.12)
Full time work	502 (36.4)	137 (21.4)		555 (34.3)	84 (20.9)		565 (34.6)	74 (19.1)		558 (33.9)	81 (21.8)	
Part time work	266 (19.3)	104 (16.2)		303 (18.7)	67 (16.7)		314 (19.2)	56 (14.5)		311 (18.9)	59 (15.9)	
Casual work	84 (6.1)	55 (8.6)		95 (5.9)	44 (10.9)		107 (6.6)	32 (8.3)		112 (6.8)	27 (7.3)	
Looking for work	109 (7.9)	76 (11.9)		130 (8)	55 (13.7)		139 (8.5)	46 (11.9)		142 (8.6)	43 (11.6)	
Not in paid workforce	417 (30.3)	269 (42)		534 (33)	152 (37.8)		507 (31.1)	179 (46.3)		524 (31.8)	162 (43.5)	
*Marital status*			< 0.001* (0.16)			< 0.001* (0.15)			< 0.001* (0.16)			< 0.001* (0.15)
Never married	385 (27.9)	199 (31)		434 (26.8)	150 (37.3)		459 (28.1)	125 (32.3)		464 (28.2)	120 (32.3)	
Married	652 (47.3)	212 (33.1)		752 (46.5)	112 (27.9)		755 (46.3)	109 (28.2)		756 (45.9)	108 (29)	
De facto (Opposite sex)	144 (10.4)	76 (11.9)		171 (10.6)	49 (12.2)		167 (10.2)	53 (13.7)		169 (10.3)	51 (13.7)	
De facto (Same sex)	21 (1.5)	8 (1.2)		21 (1.3)	8 (2)		24 (1.5)	5 (1.3)		24 (1.5)	5 (1.3)	
Separated/Divorced/Widowed	176 (12.8)	146 (22.8)		239 (14.8)	83 (20.6)		227 (13.9)	95 (24.5)		234 (14.2)	88 (23.7)	
*Highest qualification*			< 0.001* (0.10)			0.022* (0.07)			0.001* (0.09)			0.022* (0.07)
Less than year 12	211 (15.3)	116 (18.1)		260 (16.1)	67 (16.7)		251 (15.4)	76 (19.6)		257 (15.6)	70 (18.8)	
Year 12 or equivalent	290 (21)	131 (20.4)		336 (20.8)	85 (21.1)		341 (20.9)	80 (20.7)		347 (21.1)	74 (19.9)	
Apprenticeship/ certificate/diploma	437 (31.7)	245 (38.2)		526 (32.5)	156 (38.8)		534 (32.7)	148 (38.2)		541 (32.8)	141 (37.9)	
University degree	440 (31.9)	149 (23.2)		495 (30.6)	94 (23.4)		506 (31)	83 (21.4)		502 (30.5)	87 (23.4)	
*Financial manageability*			< 0.001* (0.25)			< 0.001* (0.23)			< 0.001* (0.25)			< 0.001* (0.22)
It is difficult all of the time	206 (14.9)	224 (34.9)		275 (17)	155 (38.6)		273 (16.7)	157 (40.6)		286 (17.4)	144 (38.7)	
It is difficult some of the time	522 (37.9)	244 (38.1)		611 (37.8)	155 (38.6)		625 (38.3)	141 (36.4)		627 (38.1)	139 (37.4)	
It is not too bad	548 (39.8)	152 (23.7)		618 (38.2)	82 (20.4)		619 (37.9)	81 (20.9)		620 (37.6)	80 (21.5)	
It is easy	102 (7.4)	21 (3.3)		113 (7)	10 (2.5)		115 (7)	8 (2.1)		114 (6.9)	9 (2.4)	
*Health care card*			< 0.001* (0.10)			0.001* (0.07)			< 0.001* (0.08)			< 0.001* (0.11)
Yes	621 (45.1)	218 (34)		701 (43.4)	138 (34.3)		710 (43.5)	129 (33.3)		726 (44.1)	113 (30.4)	
No				916 (56.6)	264 (65.7)							
*Private health insurance*			< 0.001* (0.11)			< 0.001* (0.12)			< 0.001* (0.12)			< 0.001* (0.08)
Yes	650 (47.2)	378 (18.7)		777 (48.1)	251 (62.4)		785 (48.1)	243 (62.8)		807 (49)	221 (59.4)	
No	728 (52.8)	263 (41)		840 (51.9)	151 (37.6)		847 (51.9)	144 (37.2)		840 (51)	151 (40.6)	

Note. *p < .05. The Australian health care card is a concession card providing discounted medicines and services to eligible low income patients and is funded by the Australian Federal Government. ARIA is the Accessibility/Remoteness Index of Australia supported and utilised by the Commonwealth Dept of Health and the Australian Bureau of Statistics. ACT is the Australian Capital Territory

Age and remoteness of residence was significantly associated (*p* < .05) with having either any mental health disorder, anxiety or a mood disorder, but *not* with having any other mental health disorder (not anxiety or mood). Participants who were 60 years and over reported any of these diagnoses less frequently than all other age groups. Compared to those living in outer regional/remote areas, participants living in a major city or inner regional area more frequently reported having any MHD.

### Health related characteristics

Table 2 summarises the results of Chi-square tests of association between MHD, physical health diagnosis and number of chronic conditions. Each category of MHD had large significant associations (*p* < .01) with each comparable MHD category (Cramer’s V between 0.45 and 0.58). Participants reporting an anxiety disorder, compared to those without, were more likely to have either a mood disorder diagnosis or any other MHD. Similarly, compared to participants without a mood disorder, those reporting a mood disorder diagnosis were more likely to have any other MHD.

**Table 2:** Associations between comorbid mental and physical health diagnosis with type of mental health disorder

Sociodemographic show characteristic	Any mental health disorder n (%)			Anxiety disorder n (%)			Mood disorder n (%)			Other mental health disorder n (%)		
	No (*n* = 1378)	Yes (*n* = 641)	*p*-value (Cramer’s V)	No (*n* = 1617)	Yes (*n* = 402)	*p*-value (Cramer’s V)	No (*n* = 1632)	Yes (*n* = 387)	*p*-value (Cramer’s V)	No (*n* = 1645)	Yes (*n* = 371)	*p*-value (Cramer’s V)
*Mental health diagnosis*												
Anxiety disorder	-	-	-	-	-	-	142 (8.7)	260 (67.2)	< 0.001* (0.58)	186 (11.3)	216 (58.1)	< 0.001* (0.45)
Mood disorder	-	-	-	127 (7.9)	260 (64.7)	< 0.001* (0.58)	-	-	-	177 (10.7)	210 (56.5)	< 0.001* (0.45)
Other mental health diagnosis	-	-	-	156 (9.6)	216 (53.7)	< 0.001* (0.45)	162 (9.9)	210 (54.3)	< 0.001* (0.45)	-	-	-
*Physical illness diagnosis*												
Cancer	86 (6.2)	46 (7.2)	0.429	112 (6.9)	20 (5)	0.157	105 (6.4)	27 (7)	0.698	103 (6.3)	29 (7.8)	0.277
Cardiovascular	248 (18)	197 (30.7)	< 0.001* (0.14)	328 (20.3)	117 (29.1)	< 0.001* (0.08)	320 (19.6)	125 (32.3)	< 0.001* (0.12)	331 (20.1)	114 (30.6)	< 0.001* (0.10)
Diabetes	104 (7.5)	72 (11.2)	0.006* (0.06)	140 (8.7)	36 (9)	0.850	135 (8.3)	41 (10.6)	0.145	130 (7.9)	46 (12.4)	0.006* (0.06)
Female reproductive	41 (3)	71 (11.1)	< 0.001* (0.17)	64 (4)	48 (11.9)	< 0.001* (0.14)	62 (3.8)	50 (12.9)	< 0.001* (0.16)	66 (4)	46 (12.4)	< 0.001* (0.14)
Gastrointestinal	124 (9)	169 (26.4)	< 0.001* (0.23)	192 (11.9)	101 (25.1)	< 0.001* (0.15)	180 (11)	113 (29.2)	< 0.001* (0.20)	188 (11.4)	105 (28.2)	< 0.001* (0.19)
Male reproductive	31 (2.2)	17 (2.7)	0.581	42 (2.6)	6 (1.5)	0.193	37 (2.3)	11 (2.8)	0.504	37 (2.2)	11 (3)	0.417
Musculoskeletal	169 (12.3)	148 (23.1)	< 0.001* (0.14)	237 (14.7)	80 (19.9)	0.010* (0.06)	213 (13.1)	104 (26.9)	< 0.001* (0.15)	222 (13.5)	95 (25.5)	< 0.001* (0.13)
Respiratory	172 (12.5)	181 (28.2)	< 0.001* (0.19)	247 (15.3)	106 (26.4)	< 0.001* (0.12)	231 (14.2)	122 (31.5)	< 0.001* (0.18)	236 (14.3)	117 (31.5)	< 0.001* (0.18)
Other chronic illness	92 (6.7)	56 (8.7)	0.098	112 (6.9)	36 (9)	0.162	107 (6.6)	41 (10.6)	0.006* (0.06)	113 (6.9)	35 (9.4)	0.089
*Number of chronic conditions*			< 0.001* (0.67)			< 0.001* (0.55)			< 0.001* (0.62)			< 0.001* (0.59)
0	705	0		705	0		705	0		705	0	
1	361 (26.2)	94 (14.7)		415 (25.7)	40 (10)		435 (26.7)	20 (5.2)		421 (25.6)	34 (9.1)	
2	169 (12.3)	115 (17.9)		210 (13)	74 (18.4)		225 (13.8)	59 (15.2)		244 (14.8)	40 (10.8)	
3	77 (5.6)	107 (16.7)		117 (7.2)	67 (16.7)		123 (7.5)	61 (15.8)		116 (7)	68 (18.3)	
4	39 (2.8)	106 (16.5)		81 (5)	64 (15.9)		72 (4.4)	73 (18.9)		80 (4.9)	65 (17.5)	
5 or more	27 (2)	219 (34.2)		89 (5.5)	157 (39.1)		72 (4.4)	174 (45)		81 (4.9)	165 (44.4)	

Note. * *p* < .05

A physical health diagnosis of a cardiovascular, female reproductive, gastrointestinal, musculoskeletal or respiratory condition were all statistically significantly associated with each category of MHD (*p* < .05). However, the strength of the associations varied according to the specific category of physical illness diagnosis and MHD. For example, the association between musculoskeletal conditions and anxiety disorders was negligible (Cramer’s V = 0.06), compared to a moderate association between gastrointestinal disorders and any MHD (Cramer’s V = 0.23). Participants reporting each of these physical conditions were more likely to report any MHD, anxiety or mood disorder, compared to those without each condition.

Participants with a diabetes diagnosis were more likely to report having any MHD or other MHD, although the association was of negligible significance (*p* = .006, Cramer’s V = 0.06). Participants reporting other physical illnesses were significantly (*p* > .01) more likely to report a mood disorder, although the association was negligible (Cramer’s V = 0.06).

Number of chronic conditions had large significant associations with all categories of MHD (*p* > .001, Cramer’s V between 0.55 and 0.67). People with five or more illnesses had the highest reported rates of all MHD categories, including any mental health disorder (34.2 %), anxiety (39.1 %), mood (45 %) or other MHD (44.4 %).

### Prevalence of conventional and complementary health care use

All forms of conventional health care use (i.e., medical doctor, allied health and pharmaceuticals) were significantly associated with each MHD category (*p* < .001); with the strength of associations ranging between negligible (Cramer’s V = 0.05 for physiotherapist and mood disorder) to large (Cramer’s V = 0.46 for psychologist/counsellor and any MHD). Within the medical doctor category, participants most frequently consulted a general practitioner for all categories of MHD (over 96 %), followed by hospital doctors, who were consulted by between 41.8 and 47.3 % of participants. Within the allied health category, most people consulted a pharmacist across all types of MHD (88–89.9 %), with the lowest consultation rates reported for community nurses (15.6 to 19.9 %). The highest use of pharmaceutical medicines related to prescription use across all MHD categories, including any mental health disorder (90.6 %), anxiety (92 %), mood (92.8 %) and other (91.1 %). See Table 3 for summary statistics.

**Table 3:** Prevalence of conventional and CM health service and treatment use associated with mental health diagnosis

Type of health care	Any mental health disorder n (%)			Anxiety disorder n (%)			Mood disorder n (%)			Other mental health disorder n (%)		
	No (*n* = 1378)	Yes (*n* = 641)	*P*-value (Cramer’s V)	No (*n* = 1617)	Yes (*n* = 402)	*P*-value (Cramer’s V)	No (*n* = 1632)	Yes (*n* = 387)	*P*-value (Cramer’s V)	No (*n* = 1647)	Yes (*n* = 372)	*P*-value (Cramer’s V)
*Conventional health care*												
*Medical doctor*												
Hospital doctor	300 (21.8)	268 (41.8)	< 0.001* (0.21)	393 (24.3)	175 (43.5)	< 0.001* (0.17)	393 (24.3)	175 (43.5)	< 0.001* (0.18)	392 (23.8)	176 (47.3)	< 0.001* (0.20)
General practitioner	1137 (82.5)	619 (96.6)	< 0.001* (0.194)	1367 (84.5)	389 (96.8)	< 0.001* (0.15)	1382 (84.7)	374 (96.8)	< 0.001* (0.14)	1396 (84.8)	360 (96.8)	< 0.001* (0.14)
Specialist doctor	467 (33.9)	372 (58)	< 0.001* (0.23)	618 (38.2)	221 (55)	< 0.001* (0.14)	618 (38.2)	221 (55)	< 0.001* (0.18)	607 (36.9)	232 (62.4)	< 0.001* (0.20)
*Allied health*												
Pharmacist	980 (71.1)	564 (88)	< 0.001* (0.19)	1186 (73.3)	358 (89.1)	< 0.001* (0.15)	1196 (73.3)	348 (89.9)	< 0.001* (0.15)	1211 (73.5)	333 (89.5)	< 0.001* (0.15)
Physiotherapist	263 (19.1)	172 (26.8)	< 0.001* (0.09)	330 (20.4)	105 (26.1)	0.013* (0.06)	334 (20.5)	101 (26.1)	0.015* (0.05)	329 (20)	106 (28.5)	< 0.001* (0.08)
Psychologist/ counsellor	111 (8.1)	307 (47.9)	< 0.001* (0.46)	196 (12.1)	222 (55.2)	< 0.001* (0.43)	199 (12.2)	219 (56.6)	< 0.001* (0.43)	230 (14)	188 (50.5)	< 0.001* (0.35)
Community nurse	104 (7.5)	100 (15.6)	< 0.001* (0.12)	139 (8.6)	65 (16.2)	< 0.001* (0.10)	143 (8.8)	61 (15.8)	< 0.001* (0.09)	130 (7.9)	74 (19.9)	< 0.001* (0.15)
*Pharmaceuticals*												
Over-the-counter	853 (61.9)	496 (77.4)	< 0.001* (0.15)	1045 (64.6)	304 (75.6)	< 0.001* (0.09)	1046 (64.1)	303 (78.3)	< 0.001* (0.12)	1058 (64.2)	291 (78.2)	< 0.001* (0.12)
Prescription only	921 (66.8)	581 (90.6)	< 0.001* (0.25)	1132 (70)	370 (92)	< 0.001* (0.20)	1143 (70)	359 (92.8)	< 0.001* (0.21)	1163 (70.6)	339 (91.1)	< 0.001* (0.18)
*Complementary health care*												
*CM practitioner*												
Acupuncturist	92 (6.7)	67 (10.5)	0.003* (0.07)	122 (7.5)	37 (9.2)	0.269	125 (7.7)	34 (8.8)	0.460	114 (6.9)	45 (2.2)	0.001* (0.07)
Aromatherapist	46 (3.3)	33 (5.1)	0.051 (0.04)	59 (3.6)	20 (5.0)	0.22	62 (3.8)	17 (4.4)	0.588	51 (3.1)	28 (7.5)	< 0.001* (0.09)
Chiropractor	151 (11)	103 (16.1)	0.001* (0.07)	196 (12.1)	58 (14.4)	0.212	195 (11.9)	59 (15.2)	0.079	180 (10.9)	74 (19.9)	< 0.001* (0.11)
Homeopath	39 (2.8)	29 (4.5)	0.05* (0.044)	51 (3.2)	17 (4.2)	0.285	57 (3.5)	11 (2.8)	0.524	42 (2.6)	26 (7)	< 0.001* (0.10)
Massage therapist	265 (19.2)	153 (23.9)	0.017* (0.05)	320 (19.8)	98 (24.4)	0.042* (0.05)	329 (20.2)	89 (23)	0.215	323 (19.6)	95 (25.5)	0.011* (0.06)
Naturopath	68 (4.9)	58 (9)	< 0.001* (0.08)	92 (5.7)	34 (8.5)	0.04* (0.05)	100 (6.1)	26 (6.7)	0.666	81 (4.9)	45 (12.1)	< 0.001* (0.12)
Osteopath	62 (4.5)	48 (7.5)	0.006* (0.06)	80 (4.9)	30 (7.5)	0.047* (0.04)	91 (5.6)	19 (4.9)	0.604	77 (4.7)	33 (8.9)	0.001* (0.07)
TCM practitioner	67 (4.9)	40 (6.2)	0.198	85 (5.3)	22 (5.5)	0.863	88 (5.4)	19 (4.9)	0.703	75 (4.6)	32 (8.6)	0.002* (0.07)
Western herbalist	45 (3.3)	31 (4.8)	0.084	57 (3.5)	19 (4.7)	0.257	59 (3.6)	17 (4.4)	0.470	54 (3.3)	22 (5.9)	0.016* (0.05)
Yoga teacher	113 (8.2)	67 (10.5)	0.098	134 (8.3)	46 (11.4)	0.047* (0.04)	142 (8.7)	38 (9.8)	0.488	135 (8.2)	45 (12.1)	0.017* (0.05)
Other CM practitioner	6 (0.4)	^	0.918	8 (0.5)	^	0.508	7 (0.4)	^	0.816	7 (0.4)	^	0.768
Any CM practitioner	454 (32.9)	272 (42.4)	< 0.001* (0.09)	558 (34.5)	168 (41.8)	0.006* (0.06)	567 (34.7)	159 (41.1)	0.019* (0.05)	558 (33.9)	168 (45.2)	< 0.001* (0.09)
*CM products and practices*												
Aromatherapy oils	133 (9.7)	91 (14.2)	0.002* (0.07)	159 (9.8)	65 (16.2)	< 0.001* (0.08)	176 (10.8)	48 (12.4)	0.362	161 (9.8)	63 (16.9)	< 0.001* (0.09)
Flower essences	102 (7.4)	49 (7.6)	0.847	119 (7.4)	32 (8.0)	0.682	128 (7.8)	23 (5.9)	0.201	119 (7.2)	32 98.6)	0.362
Homeopathy	97 (7)	41 (6.4)	0.595	111 (6.9)	27 (6.7)	0.916	120 (7.4)	18 (4.7)	0.058	110 (6.7)	28 (7.5)	0.558
Vitamin/mineral supplements	614 (44.6)	352 (54.9)	< 0.001* (0.10)	746 (46.1)	220 (54.7)	0.002* (0.07)	759 (46.5)	207 (53.5)	0.013* (0.055)	763 (46.3)	203 (54.6)	0.004* (0.06)
Western or Chinese herbal medicines	133 (9.7)	58 (9)	0.666	154 (9.5)	37 (9.2)	0.845	162 (9.9)	29 (7.5)	0.141	152 (9.2)	39 (10.5)	0.455
Any CM product	651 (47.2)	365 (56.9)	< 0.001* (0.09)	786 (48.6)	230 (57.2)	0.002* (0.07)	801 (49.1)	215 (55.6)	0.022* (0.05)	806 (48.9)	210 (56.5)	0.009* (0.06)
Relaxation/ meditation	183 (13.3)	137 (21.4)	< 0.001* (0.10)	226 (14)	94 (23.4)	< 0.001* (0.10)	232 (14.2)	88 (22.7)	< 0.001* (0.09)	232 (14.1)	88 (23.7)	< 0.001* (0.10)
Yoga, Tai Chi or Qi Gong	158 (11.5)	79 (12.3)	0.577	185 (11.4)	52 (12.9)	0.405	200 (12.3)	37 (9.6)	0.139	186 (11.3)	51 (13.7)	0.191
Any CM practice	227 (16.5)	150 (23.4)	< 0.001* (0.08)	278 (17.2)	99 (24.6)	0.001* (0.08)	285 (17.5	92 (23.8)	0.004* (0.06)	281 (17.1)	96 (25.8)	< 0.001* (0.09)
Any CM treatment (product or practice)	682 (49.5)	385 (60.1)	< 0.001* (0.10)	826 (51.1)	241 (60)	0.001* (0.07)	838 (51.3)	229 (59.2)	0.006* (0.06)	842 (51.1)	225 (60.5)	0.001* (0.07)
Any CM use (product or practice or practitioner consultation)	818 (59.4)	455 (71)	< 0.001* (0.11)	991 (61.3)	282 (70.1)	0.001* (0.07)	1002 (61.4)	271 (70)	0.002* (0.07)	1006 (61.1)	267 (71.8)	< 0.001* (0.09)

Note. *p < .05. ^ Cell count excluded if < 5. The CM practitioner category Traditional/spiritual healer was excluded as all cell counts were < 5. Hospital doctors refers to a range of medical practitioners located in the tertiary care sector who treat patients admitted or referred to hospital

Complementary health care use was categorised as: CM practitioner consultations, and CM products and practices (i.e., non-ingestible treatments such as massage therapies, yoga and Tai Chi). Consulting with *any* CM practitioner in the previous 12 months was significantly associated with each MHD category (*p* < .001). The significance and strength of associations for each type of CM practitioner varied across MHD categories (see Table 3). The largest proportion of participants consulted a massage therapist for any MHD (23.9 %), anxiety (24.4 %) and other MHD (25.5 %). In contrast, homeopaths were consulted the least for any MHD (4.5 %), anxiety (4.2 %) and mood (4.2 %) disorder, and acupuncturists consulted the least for other MHD (2.2 %). There were no statistically significant associations between any specific type of CM practitioner and people reporting a mood disorder diagnosis.

Use of CM practice or product was significantly associated with all MHD categories (*p* < .05). The significance and strength of associations, and the prevalence of use, for each type of CM practice or product varied by MHD category (see Table 3). Vitamin or mineral supplements had the highest usage rates across all MHD, including any MHD (54.9 %), anxiety (54.7 %), mood (53.5 %), and other MHD (54.6 %). Relaxation techniques had the highest prevalence of use of the CM practices, which were used by 21.4–23.7 % of participants across all MHD categories.

### Out-of-pocket expenditure

On average in the previous 12 months, the total self-reported OOP expenditure on health care by each participant with a MHD was AUD$ 1030.06 (US$ 767.12). This extrapolated to estimated OOP costs of AUD$ 5,752,019,906 (US$ 4,284,028,624) for health care used by adults with a MHD in Australia (*n* = 5,584,169) with AUD$ 4,568,267,421 (US$ 3,398,293,672) for conventional health care practitioners and medicines, and AUD$ 1,183,752,486 (US$ 880,729,891) for complementary medicine practitioners, products and practices.

The OOP expenditure on each type of health service and treatment in the previous 12 months is reported in Table 4. Participants with a MHD, compared to those without, spent significantly more on both over-the-counter (OTC) pharmaceutical medicines (*U* = 377,595, *z* = 4.01, *p* < .000) and prescription medicines (*U* = 404,783, *z* = 6.76, *p* < .000). For those consulting with conventional health practitioners, those with a MHD spent significantly more than those without a MHD on specialist doctors (*U* = 424,837, *z* = 5.89, *p* < .000), hospital doctors (*U* = 389,032, *z* = 2.97, *p* < .000) and counsellors/psychologists (*U* = 428,171, *z* = 9.41, *p* < .000).

**Table 4:** Out-of-pocket expenditure on type of health service and treatment used in the previous 12 months

Type of treatment show or service used	Total annual expenses MHD participants (*n* = 641)	Estimated total annual expenses for Australian population with a MHD (AUD) (*n* = 5,584,169.29)^a^	Mean/median annual expense			
			MHD participants (*n* = 641)		No-MHD participants (*n* = 1378)	
			Mean	Median	Mean	Median
*Pharmaceuticals*						
Prescription-only	93,237	812,246,608	145.46	30.00*	82.38	12.00*
Over-the-counter	34,853	303,629,840	54.37	20.00*	32.62	10.00*
Total	128,090	1,115,876,448	199.83	60.00	115.00	30.00
*CM products*						
Vitamins/Mineral Supplements	32,387	282,146,905	50.53	5.00	40.57	0.00
Aromatherapy oils	4,579	39,890,657	7.14	0.00	4.21	0.00
Western/Chinese herbal medicine	4,307	37,521,088	6.72	0.00	5.24	0.00
Homeopathy	4,058	35,351,886	6.33	0.00	2.31	0.00
Flower Essences	2,369	20,670,152	3.70	0.00	1.99	0.00
Total	47,580	415,580,687	74.34	10.00	54.48	0.00
*CHC practitioner*						
General practitioner	203,829	1,775,687,082	317.99	0.00	43.10	0.00
Specialist doctor	89,775	782,086,866	140.05	0.00*	111.52	0.00*
Hospital doctor	47,855	416,896,133	74.66	0.00*	17.88	0.00*
Counsellor/Psychologist	38,287	333,543,042	59.73	0.00*	11.08	0.00*
Community nurse	2,825	24,610,418	4.41	0.00	3.16	0.00
Physiotherapist	13,725	119,567,431	21.41	0.00	19.04	0.00
Total	396,2956	3,452,390,973	618.25	60.00	205.80	25.00
*CM practitioner*						
Massage therapist	17,853	155,772,147	27.90	0.00	16.77	0.00
Chiropractor	15,418	134,316,259	24.05	0.00	12.57	0.00
Yoga teacher	3,463	30,168,453	5.40	0.00	4.74	0.00
Acupuncturist	7,431	64,837,441	11.61	0.00	5.13	0.00
Naturopath	10,169	88,588,795	15.86	0.00*	4.02	0.00*
Osteopath	4,334	37,756,302	6.76	0.00	3.99	0.00
TCM practitioner	3,537	30,813,115	5.52	0.00	4.07	0.00
Aromatherapist	8,992	78,335,180	14.03	0.00	2.10	0.00
Homeopath	3,268	28,469,681	5.10	0.00	3.36	0.00
Western Herbalist	3,731	32,503,176	5.82	0.00	2.77	0.00
Total	70,532	681,560,548	110.38	0.00	59.41	0.00
*CM practices*						
Yoga/tai chi	4,076	35,508,696	6.36	0.00*	6.58	0.00*
Relaxation/ meditation	5,866	51,102,554	9.15	0.00	3.36	0.00
Total	9,942	86,611,250	15.51	0.00	9.95	0.00

Note. *AUD* Australian dollars, *MHD* mental health disorder, *CHC* conventional health care, *CM* complementary medicine, *TCM* traditional Chinese medicine. ^**a**^The estimated number of the Australian population with a MHD was calculated based on Australian census figures in year 2016 for Australian adults aged 20 years or above (*n* = 17,615,676) and the reported prevalence of mental health disorders in this study (31.7 %). *Indicates a significant difference between median expenses of MHD and no-MHD groups (*p* < .05)

For those consulting with a CM practitioner, a significant difference in expenses was only found for participants consulting with a naturopath; those with a MHD spent significantly more on consultations than those without a MHD, *U* = 66,122, *z* = 2.53, *p* = .01. For those who used CM products, participants with a MHD spent significantly less on yoga, Tai Chi or Qi Gong compared to those without a diagnosis, *U* = 14,319, *z* = -2.86, *p* < .000. There was no significant difference in median expenditure on each type of CM product between participants with or without a MHD.

Table 5 presents the results of the hierarchical multiple regression predicting total health care expenses from MHD diagnosis. The full model including relevant sociodemographic variables, chronic physical illness and MHD (Model 3) was statistically significant (*R*^2^ = 0.038, *F*(11, 2001) = 7.111, *p* < .0001, adjusted *R*^2^ = 0.033); accounting for less than 4 % of the variance in total health care expenditure. Employment status, private health insurance, chronic physical illness and MHD were all significant predictors of total health care expenses in the final model.

**Table 5:** Hierarchical multiple regression predicting total health care expenses

	Total health care expenses					
	Model 1		Model 2		Model 3	
Variable	B	ß	B	ß	B	ß
Constant	1058.57**		947.58**		557.54*	
Gender	-44.28	-0.02	-26.49	-0.01	-4.91	0.00
Age	-3.04	0.00	-28.13	-0.04	-16.31	-0.02
ARIA+	14.96	0.01	11.24	0.01	12.88	0.01
Marital status	18.79	0.02	15.70	0.02	9.65	0.01
Highest qualification	-24.08	-0.02	-25.55	-0.03	-26.49	-0.03
Financial manageability	-1.40	0.00	-0.64	0.00	25.00	0.02
Employment status	-22.80	-0.04	-32.53	-0.05	-41.52*	-0.07
Private health insurance	-229.92**	-0.11	-215.56**	-0.10	-221.35**	-0.10
Health care card	-50.84	-0.02	-36.40	-0.02	-25.24	-0.01
Chronic physical illness			288.46**	0.13	248.56**	0.12
Mental health disorder					244.56**	0.11
*R*^2^	0.012		0.028		0.038	
*F*	2.72*		5.74**		7.11**	
Δ*R*^2^	0.012*		0.016**		0.010**	

Note. *p < .05, **p < .001. B = Unstandardised regression coefficient, ß **=** Standardised regression coefficient, Δ*R*^2^ = squared part correlations. The Australian health care card is a concessions card providing discounted medicines and services to eligible low income patients and is funded by the Australian Federal Government. ARIA is the Accessibility/Remoteness Index of Australia supported and utilised by the Commonwealth Dept of Health and the Australian Bureau of Statistics. ACT is the Australian Capital Territory

### Predictors of type of health practitioner use

The logistic regression models predicting each type of health practitioner visited were statistically significant (*p* < .001; see Table 6). Gender, employment status, financial management, private health insurance, health care card, MHD and chronic physical illness diagnosis were all significant predictors of general practitioner consultations (*p* < .05). Age, qualification level, private health insurance, MHD and chronic physical illness significantly predicted (*p* < .05) specialist doctor consultations. For psychologist/counsellor consultations, six predictor variables were statistically significant (*p* < .05): age, marital status, employment status, health care card, MHD and chronic physical illness. CM practitioner consultations were significantly (*p* < .05) predicted by gender, age, employment status, private health insurance, MHD, and chronic physical illness.

**Table 6:** Logistic regressions predicting likelihood of consulting with each type of health practitioner

Characteristic	General practitioner			Specialist doctor			Psychologist or counsellor			CM practitioner (any)		
	Odds ratio	95 % CI	*p*-value	Odds ratio	95 % CI	*p*-value	Odds ratio	95 % CI	*p*-value	Odds ratio	95 % CI	*p*-value
*Gender (male)*	0.67	0.478, 0.857	0.003*	1.03	0.827, 0.827	0.800	1.16	0.875, 1.533	0.305	0.71	0.573, 0.878	0.002*
*Age*												
18–29	-	-	< 0.001**	-	-	< 0.001**	-	-	< 0.001**	-	-	< 0.001**
30–39	1.11	0.216, 0.563	< 0.001**	0.90	0.634, 1.278	0.556	0.80	0.533, 1.213	0.298	0.90	0.652, 1.252	0.543
40–49	1.02	0.271, 0.792	0.005*	1.07	0.752, 1.508	0.723	0.80	0.529, 1.215	0.298	0.73	0.526, 1.018	0.064
50–59	1.13	0.279, 0.793	0.005*	0.66	0.453, 0.972	0.035	0.39	0.243, 0.639	< 0.001**	0.54	0.373, 0.775	0.001*
60 and over	1.55	0.375, 1.140	0.134	1.37	0.938, 2.001	0.104	0.27	0.160, 0.447	< 0.001**	0.40	0.273, 0.581	< 0.001**
*ARIA+*												
Major cities	-	-	0.108	-	-	0.156	-	-	0.433	-	-	0.658
Inner regional	0.72	0.488, 1.054	0.091	0.79	0.602, 1.030	0.082	0.87	0.618, 1.215	0.406	0.89	0.679, 1.160	0.383
Outer regional‎/Remote‎	0.67	0.396, 1.115	0.122	0.82	0.568, 1.170	0.267	0.76	0.467, 1.232	0.264	0.93	0.648, 1.328	0.682
*Marital status*												
Never married	-	-	0.455	-	-	0.089	-	-	0.012*	-	-	0.109
Married	1.22	0.816, 1.822	0.333	1.18	0.884, 1.568	0.265	1.08	0.753, 1.560	0.665	1.14	0.861, 1.500	0.367
De facto (Opposite sex)	1.46	0.871, 2.431	0.152	0.96	0.658, 1.392	0.819	1.10	0.700, 1.717	0.688	0.72	0.499, 1.035	0.076
De facto (Same sex)	2.12	0.460, 9.715	0.336	0.90	0.386, 2.101	0.808	4.40	1.804, 10.720	0.001*	1.43	0.644, 3.163	0.381
Separated/ Divorced/Widowed	1.49	0.831, 2.680	0.180	1.58	1.103, 2.251	0.012	1.45	0.934, 2.242	0.098	1.14	0.798, 1.626	0.472
*Highest level of qualification*												
Less than Year 12	-	-	0.270	-	-	0.028*	-	-	0.924	-	-	0.178
Year 12 or equivalent	1.37	0.834, 2.264	0.212	0.81	0.572, 1.140	0.224	1.06	0.680, 1.656	0.794	0.92	0.650, 1.307	0.647
Trade/apprenticeship/ certificate/diploma	1.35	0.850, 2.130	0.205	1.06	0.779, 1.432	0.726	1.12	0.751, 1.670	0.579	1.13	0.827, 1.543	0.443
University degree	1.02	0.623, 1.653	0.954	1.29	0.919, 1.809	0.141	1.15	0.733, 1.816	0.537	1.26	0.898, 1.767	0.181
*Manage financially*												
It is impossible/difficult all the time	-	-	0.048*	-	-	0.173	-	-	0.077	-	-	0.735
It is difficult some of the time	0.80	0.520, 1.216	0.290	0.78	0.587, 1.022	0.071	0.79	0.571, 1.088	0.148	1.15	0.872, 1.514	0.323
It is not too bad	0.97	0.617, 1.513	0.881	0.72	0.537, 0.970	0.031	0.63	0.434, 0.907	0.013	1.14	0.852, 1.532	0.373
It is easy	0.46	0.243, 0.867	0.016*	0.74	0.463, 1.195	0.221	0.95	0.511, 1.769	0.872	1.24	0.776, 1.969	0.372
*Employment status*												
Full time work	-	-	0.048	-	-	0.300	-	-	0.002*	-	-	< 0.001**
Part time work	0.92	0.613, 1.389	0.699	1.21	0.889, 1.648	0.225	1.35	0.901, 2.012	0.147	0.84	0.626, 1.113	0.218
Casual/temp work	1.27	0.688, 2.327	0.449	1.04	0.668, 1.625	0.856	1.81	1.088, 3.017	0.022*	0.69	0.456, 1.041	0.077
Looking for work	0.61	0.373, 0.994	0.047	0.85	0.561, 1.300	0.462	1.89	1.156, 3.078	0.011*	0.30	0.194, 0.458	< 0.001**
Not in the paid workforce	1.38	0.877, 2.180	0.163	1.25	0.921, 1.692	0.153	0.85	0.561, 1.284	0.438	0.48	0.353, 0.642	< 0.001**
*Private health insurance (yes)*	0.72	0.525, 0.992	0.045	0.59	0.473, 0.739	< 0.001**	0.77	0.578, 1.035	0.084	0.58	0.466, 0.717	< 0.001**
*Health care card (yes)*	0.71	0.524, 0.952	0.022	0.86	0.689, 1.060	0.153	0.70	0.526, 0.924	0.012	0.90	0.734, 1.114	0.345
*Chronic physical illness (yes)*	5.86	4.065, 8.436	< 0.001**	4.11	3.308, 5.112	< 0.001**	1.88	1.417, 2.497	< 0.001**	1.69	1.367, 2.096	< 0.001**
*Mental health diagnosis (yes)*	4.41	2.741, 7.095	< 0.001**	2.47	1.966, 3.091	< 0.001**	9.93	7.512, 13.133	< 0.001**	1.70	1.354, 2.124	< 0.001**
Constant	3.62		< 0.001**	0.31		< 0.001**	0.12		< 0.001**	0.99		0.954
Model fit												
χ^2^ (df)	306.33 (25)			428.85 (25)			523.42 (25)			247.24 (25)		
Nagelkerke *R*^2^	0.262			0.258			0.358			0.158		
P-value	< 0.001**			< 0.001**			< 0.001**			< 0.001**		

Note. *p < .05, **p < .001. The Australian health care card is a concessions card providing discounted medicines and services to eligible low income patients and is funded by the Australian Federal Government. ARIA is the Accessibility/Remoteness Index of Australia supported and utilised by the Commonwealth Dept of Health and the Australian Bureau of Statistics. ACT is the Australian Capital Territory

Males were less likely to see a general practitioner (*OR* = 0.666, 95% CI [0.490, 0.905]) or any CM practitioner than females (*OR* = 0.709, 95% CI [0.573, 0.878]). Being 50–59 years old was associated with an increased likelihood of consulting with a specialist doctor. In contrast, older people aged 50–59 and 60 and over were less likely to consult a CM practitioner (*OR* = 0.538, 95% CI [0.373, 0.775]; *OR* = 0.398, 95% CI [0.273, 0.581] respectively) or a psychologist/counsellor (*OR* = 0.394, 95% CI [0.243, 0.639]; *OR* = 0.267, 95% CI [0.160, 0.447] respectively). Participants either in casual/temp work or looking for work were almost twice as likely to visit a psychologist or counsellor (*OR* = 1.812, 95% CI [1.088, 3.017]; *OR* = 1.886, 95% CI [1.156, 3.078], respectively); similarly people either looking for work or not in the workforce were less likely to visit a CM practitioner (*OR* = 0.298, 95% CI [0.194, 0.458]; *OR* = 0.476, 95% CI [0.353, 0.642], respectively). People with a MHD or chronic physical illness were more likely to consult with any health practitioner compared to those without a MHD or chronic physical illness respectively.

### Predictors of type of health treatment use

Table 7 summarises the results of the four logistic regression models predicting each type of health treatment used, which were statistically significant (*p* < .001). Prescription pharmaceutical use was significantly (*p* < .05) predicted by age, financial management, private health insurance, MHD and chronic physical illness. Gender, age, area of residence, private health insurance, MHD and chronic physical illness were significant predictors of OTC pharmaceutical use (*p* < .05). Gender, qualification level, private health insurance MHD and chronic physical illness significantly predicted CM product use (*p* < .05), whilst age, qualification level, health care card and MHD significantly predicted CM mind-body practice use (*p* < .05).

**Table 7:** Logistic regression predicting likelihood of health care product or practice use based on socio-demographics and mental health diagnosis

Characteristics	Prescription pharmaceuticals			Over-the-counter pharmaceuticals			CM products			CM mind-body practices		
	Odds ratio	95 % CI	*P*-value	Odds ratio	95 % CI	*P*-value	Odds ratio	95 % CI	*P*-value	Odds ratio	95 % CI	*P*-value
*Gender (male)*	0.79	0.619, 1.010	0.060	0.74	0.600, 0.910	0.004*	0.563	0.461, 0.687	< 0.001**	0.82	0.635, 1.063	0.136
*Age*												
18–29	-	-	0.006*	-	-	0.012*			0.443			< 0.001**
30–39	1.28	0.884, 1.866	0.190	1.38	0.984, 1.921	0.063	0.96	0.696, 1.313	0.781	0.62	0.430, 0.894	0.010*
40–49	1.06	0.727, 1.535	0.774	1.60	1.135, 2.250	0.007*	1.07	0.774, 1.464	0.700	0.54	0.367, 0784	0.001*
50–59	0.99	0.656, 1.506	0.976	0.94	0.657, 1.341	0.729	0.92	0.648, 1.293	0.616	0.55	0.359, 0.830	0.005*
60 and over	2.04	1.307, 3.182	0.002*	1.12	0.779, 1.620	0.533	1.23	0.865, 1.747	0.251	0.21	0.128, 0.346	< 0.001**
*ARIA+*												
Major cities	-	-	0.253	-	-	0.010*	-	-	0.171	-	-	0.630
Inner regional	1.23	0.890, 1.692	0.211	1.36	1.043, 1.771	0.023*	1.27	0.990, 1.620	0.060	1.16	0.841, 1.598	0.368
Outer regional‎/Remote‎	0.83	0.552, 1.257	0.383	1.56	1.078, 2.271	0.019*	1.06	0.762, 1.482	0.719	0.96	0.609, 1.507	0.851
*Marital status*												
Never married	-	-	0.161	-	-	0.802	-	-	0.161	-	-	0.154
Married	1.21	0.875, 1.669	0.250	0.98	0.739, 1.294	0.874	1.14	0.875, 1.484	0.333	0.96	0.695, 1.336	0.823
De facto (Opposite sex)	1.40	0.929, 2.096	0.108	0.92	0.650, 1.315	0.662	0.77	0.547, 1.078	0.128	1.05	0.700, 1.586	0.803
De facto (Same sex)	3.90	1.078, 14.087	0.038	1.58	0.648, 3.832	0.315	1.48	0.660, 3.316	0.341	2.32	1.015, 5.323	0.046
Separated/ Divorced/Widowed	1.19	0.768, 1.827	0.443	1.07	0.745, 1.525	0.728	1.05	0.757, 1.467	0.754	1.34	0.873, 2.052	0.181
*Highest level of qualification*												
Less than Year 12	-	-	0.706	-	-	0.177	-	-	< 0.001**	-	-	< 0.001**
Year 12 or equivalent	1.20	0.808, 1.786	0.365	1.27	0.919, 1.757	0.147	0.94	0.689, 1.292	0.717	1.11	0.695, 1.770	0.665
Trade/apprenticeship/certificate/diploma	1.22	0.849, 1.749	0.285	1.39	1.038, 1.859	0.027	1.49	1.126, 1.970	0.005*	1.48	0.970, 2.254	0.069
University degree	1.11	0.751, 1.645	0.598	1.25	0.905, 1.727	0.175	1.87	1.368, 2.559	< 0.001**	2.18	1.397, 3.390	0.001*
*Manage financially*												
It is impossible/ difficult all the time	-	-	0.054*	-	-	0.148	-	-	0.898	-	-	0.972
It is difficult some of the time	0.90	0.643, 1.255	0.530	0.88	0.667, 1.160	0.362	1.00	0.771, 1.285	0.972	0.95	0.686, 1.321	0.768
It is not too bad	0.71	0.498, 0.999	0.049*	0.73	0.548, 0.980	0.036	0.92	0.701, 1.208	0.547	0.92	0.643, 1.305	0.629
It is easy	0.55	0.325, 0.937	0.028*	0.71	0.452, 1.124	0.145	0.98	0.629, 1.513	0.913	0.94	0.532, 1.645	0.818
*Employment status*												
Full time work	-	-	0.209	-	-	0.066	-	-	0.114	-	-	0.126
Part time work	1.12	0.799, 1.556	0.521	0.94	0.701, 1.273	0.707	1.18	0.891, 1.568	0.247	0.80	0.566, 1.125	0.197
Casual/temp work	0.81	0.510, 1.279	0.363	1.25	0.799, 1.944	0.331	1.15	0.771, 1.718	0.491	0.76	0.470, 1.233	0.268
Looking for work	0.83	0.540, 1.261	0.375	0.68	0.464, 0.990	0.044	0.72	0.495, 1.047	0.086	0.63	0.391, 1.002	0.051
Not in the paid workforce	1.29	0.912, 1.825	0.150	0.77	0.573, 1.031	0.079	0.94	0.713, 1.249	0.683	0.63	0.435, 0.918	0.016
*Private health insurance (yes)*	0.62	0.483, 0.802	< 0.001**	0.79	0.634, 0.974	0.028*	0.73	0.595, 0.893	0.002*	0.79	0.602, 1.022	0.072
*Health care card (yes)*	0.88	0.69, 1.115	0.285	1.11	0.903, 1.365	0.322	1.03	0.848, 1.257	0.753	0.71	0.550, 0.915	0.008
*Chronic physical illness (yes)*	4.49	3.497, 5.760	< 0.001**	1.63	1.323, 2.014	< 0.001**	1.66	1.357, 2.019	< 0.001**	1.23	0.957, 1.592	0.105
*Mental health diagnosis (yes)*	4.05	2.944, 5.558	< 0.001**	1.85	1.464, 2.341	< 0.001**	1.40	1.128, 1.725	0.002*	1.50	1.149, 1.954	0.003*
Constant	1.16		0.619	1.41		0.171	0.71		0.158	0.37		0.001*
Model fit												
χ^2^(df)	445.16 (25)			135.04 (25)			174.45 (25)			167.06 (25)		
Nagelkerke *R*^2^	0.292			0.090			0.104			0.129		
P-value	< 0.001**			< 0.001**			< 0.001**			< 0.001**		

Note. *p < .05, **p < .001. The Australian health care card is a concessions card providing discounted medicines and services to eligible low income patients and is funded by the Australian Federal Government. ARIA is the Accessibility/Remoteness Index of Australia supported and utilised by the Commonwealth Department of Health and the Australian Bureau of Statistics. ACT is the Australian Capital Territory

Males were less likely to use OTC pharmaceuticals (*OR* = 0.739, 95% CI [0.600, 0.910]), CM products (*OR* = 0.563, 95% CI [0.461, 0.687]) or prescription pharmaceuticals (*OR* = 0.791, 95% CI [0.610, 1.010]) than males. Those 60 and over were twice as likely to use prescription pharmaceuticals (*OR* = 2.04, 95% CI [1.307, 3.182]) than those in the youngest age group (18–29 years), while those in the 40–49 age group were more likely to use OTC pharmaceuticals (*OR* = 1.598, 95% CI [1.135, 2.250]). In contrast, all older age groups were less likely to use CM mind-body practices compared to the youngest age group. Those who’s financial management was either “not too bad” or “easy”, or had private health insurance were less likely to use prescription pharmaceuticals. Those with PHI were also less likely to use OTC pharmaceuticals or CM products, while those with a health care cared were less likely to use mind-body practices. As a participant’s qualification level rose, the greater the likelihood of the participant reporting CM product or mind-body practice use. Those with a MHD were more likely to use any type of health treatment compared to those without a MHD, with prescription pharmaceuticals having the greatest increased odds of being used (*OR* = 4.045, 95% CI [2.944, 5.558]). People reporting a chronic physical illness were also more likely to use prescription or OTC pharmaceuticals or CM products, but not mind-body practices.

## Discussion

This nationally representative study provides insights into the prevalence, sociodemographic, and health-related characteristics of Australian adults reporting a MHD. The findings also shed light on conventional and CM health care use, including OOP costs, in this population. Of significance is the identification of predictors of health care use and type of health practitioner or treatment use. Having an MHD diagnosis was an important predictor of all types of health care use. This is consistent with previous research indicating that people with a mental health condition are high users of conventional health care [4, 23] or CM health care [7].

In the current study, people with a MHD were more likely to use all types of conventional health care (i.e., doctors, allied health, prescribed pharmaceutical medicines, and OTC pharmaceutical medicines). When controlling for all other variables, having a MHD was the most important predictor of consultations with psychologists/counsellors (i.e., those with an MHD were more likely to consult with them) being almost ten times more likely to consult with them than those without a MHD. We also found that having a MHD was an important predictor of all types of ingestible medicine use. Of specific interest was the finding that people with a MHD were four times more likely than those without a MHD to be using a prescription pharmaceutical medicine. These results reflect similar findings from the APC Report [4], which found that people with a MHD were high users of primary health care practitioners, particularly general practitioners and psychologists, and high users of prescribed medication. The immediate concern with such prevalent use of both complementary and pharmaceutical medicines is the potential for medicine interactions or inappropriate medicine use. Importantly, the high prevalence of comorbid chronic conditions reported by people with a MHD is likely to be driving high medicines use and polypharmacy. This is consistent with our finding that those with a chronic physical condition were more likely than those without to use all types of medicines. Collectively, multiple conventional and complementary health care practitioners involved in the care of a person with a MHD and self-prescribing of CMs, coupled with poor interprofessional communication is likely to increase the potential for medicines interactions [24]. This further supports the need for a more coordinated approach to health service provision (including the prescribing of medicines) for people living with a MHD.

The findings from our study suggest mental health consumers are using CM health services for different needs. For example, we found that although having a mood disorder was associated with consulting with at least one type of CM practitioner, it was not associated with any specific type of CM practitioner; in contrast, those with an anxiety disorder diagnosis were more likely to consult with particular types of CM practitioners such as massage therapists. Results from previous studies investigating CM use for mental health conditions are mixed [8–10]. A lack of research on this topic, inconsistent research designs and heterogeneity of variables make it difficult to draw appropriate comparisons between the results obtained from the current study and that reported in other studies. Further research is needed to provide a clearer picture of how a specific MHD may or may not influence a patient’s choice of CM health services.

Considerable investigation has been undertaken to understand how the needs of people with a mental illness can be met. Consultation with mental health services, practitioners and consumers, together with numerous government reports draw the same conclusion—the current needs of mental health consumers are not being met [4, 25, 26]. Similarly, these key stakeholders advocate for a redesign of primary health care service delivery, especially reduction of service fragmentation and a lack of seamlessness with other health care providers, whether at the practitioner or health service level. These discussions, including further research examining the merits of varying models of health care to address these unmet needs, are vital.

The results from this study show that Australians with a MHD were high users of CM and that this may be an attempt to fill, or be filling, an unmet need. Research examining the merits of integrative health care models within primary care (those that are clinically governed by a general practitioner who can act as a gatekeeper in regards to CM use and work collaboratively with CM practitioners or directly provide CM treatments) [27–29], suggests that this model has a role to play in helping to address the current issues concerning mental health care [30–32]. To date, the role of CM practitioners as a mental health resource and their role in connection with existing conventional health care service provision has not been widely discussed. Not only is further research required to comprehensively evaluate the appropriateness of this health care model, but the known public health risks related to CM use and how best to respond to these risks also necessitates national deliberation. An example of this can be drawn from a key discussion contained in the APC Report regarding OOP costs.

The APC Report states “consumers can incur sizable out of pocket costs when accessing treatment” [4, pg. 150], which reflects the findings of our study as well as other reports [5, 11, 33]. The APC acknowledges that data concerning OOP costs for consumers is limited. Furthermore, these reports do not include CM use. Accounting for the impact of CM use on OOP costs can be a complicated matter, as rebate funding via either the public (Medicare) or private systems (private health insurance) or a consumers socioeconomic status (SES; i.e., discretionary spending capacity) can have a differential impact on the decision-making process regarding choice of health care services or treatments, and in turn, overall OOP costs [6, 34]. The cost of mental health care is known to be a factor in help-seeking, obtaining treatment and support in both the short and longer term, and is correlated with subsequent mental or physical health status [35].

The results of our study found that, in general, those with a MHD did not spend more on CM. Potential reasons for this may be the absence or presence of Medicare or private health insurance rebates, or the SES of the consumer, but other factors also could be at play. Such factors may include the influence of health literacy level, personal ideology or close peers on health care choices. Our findings highlight important omissions from the current national mental health care discourse related to economic factors. These include a high amount of mental health consumer spending on CM services, practices or products that may or may not be evidence-based, as well as a perceived benefit of CM by the consumer. These are important considerations as financial decisions about health care may result in delays in help-seeking, treatment and diagnosis. More needs to be understood about the economic factors driving both conventional and CM health care choices and how OOP costs ultimately impact mental health outcomes.

Our study has several limitations that should be considered when interpreting our results. The study may be vulnerable to random error due to sampling bias; however, as the participants were nationally consistent based on gender and age [13], this is likely to have minimal impact on the outcome of our analyses. As this was an online study it may not adequately represent Aboriginal and Torres Strait Islander peoples and other population groups who have less access to the Internet. Recall bias is a possibility given the self-reported nature of the study, and the 12-month timeframe of many questions. Participant’s mental health diagnosis was also susceptible to self-report without a confirmatory diagnosis by a qualified health professional; consequently, the data is vulnerable to detection bias. In addition, the self-reported mental health diagnosis relies upon the individual being aware of a mental health condition and seeking care, and the health care capacity being in place to diagnose mental health disorders. Consequentially, the study is likely to underestimate the real mental health problem and related expenditures in the population studied. Finally, identifying the income of the study participants and collecting data on the resident to provider ratio (possibly important in accessing care) would both have provided useful additional insights to the analysis but unfortunately such information was not collected in the study.

The increasing prevalence of MHD and associated CM use in Australia warrants closer examination. The public health implications of CM use among mental health consumers requires national discussion with a diverse range of key stakeholders to ensure there is an appropriate response at the health care service level, right through to policy. Current research examining the redesign of primary health care provision should also consider whether CM practitioners and/or integrative health care service delivery models could play a role in addressing known risks associated with CM use and the unmet needs of people living with an MHD.

## Data Availability

The datasets used and/or analysed during the current study are available from the corresponding author on reasonable request.

## Abbreviations

APC: Australian Productivity Commission
CM: complementary medicine
MHD: mental health disorder
OOP: out-of-pocket
